# Implementation of a standardized surgical technique in robot-assisted restorative rectal cancer resection: a single center cohort study

**DOI:** 10.1186/s12893-022-01809-3

**Published:** 2022-10-13

**Authors:** Jacob Damgaard Eriksen, Henriette Vind Thaysen, Katrine Jøssing Emmertsen, Anders Husted Madsen, Anders Tøttrup, Charlotte Buchard Nørager, Ken Ljungmann, Niels Thomassen, Conor Patrick Delaney, Lene Hjerrild Iversen

**Affiliations:** 1 Department of Surgery, Aarhus University Hospital, Palle Juul-Jensens Boulevard 35, 8200 Aarhus N, Denmark; 2 Department of Surgery, Randers Regional Hospital, Randers, Denmark; 3 Department of Surgery, Regional Hospital West Jutland, Esbjerg, Denmark; 4 Digestive Disease and Surgery Institute, Cleveland Clinic, Florida, Weston, USA; 5 Danish Colorectal Cancer Group, Copenhagen, Denmark

**Keywords:** Rectal cancer, Surgery, Robot, Standardization, Implementation, Anastomotic leakage

## Abstract

**Background:**

Despite increasing focus on the technical performance of total mesorectal excision over recent decades, anastomotic leakage (AL) continues to be a serious complication for many patients, even in the hands of experienced surgical teams. This study describes implementation of standardized surgical technique in an effort to reduce variability, decrease the risk of anastomotic leakage, and improve associated short-term outcomes for rectal cancer patients undergoing robot-assisted restorative rectal resection (RRR).

**Methods:**

We evaluated all rectal cancer patients undergoing robot-assisted RRR at Aarhus University Hospital between 2017 and 2020. Six standardized surgical steps directed to improve anastomotic healing were mandatory for all RRR. Additional changes were made during the period with prohibition of systemic dexamethasone and limiting the use of endoscopic stapling devices.

**Results:**

The use of the full standardization, including all six surgical steps, increased from 40.3% (95% CI, 0.28–0.54) to 86.2% (95% CI, 0.68–0.95). The incidence of AL decreased from 21.0% (95% CI, 0.12–0.33) to 6.9% (95% CI, 0.01–0.23). Length of hospital stay (LOS) decreased from 6 days (range 2–50) to 5 days (range 2–26). The rate of patients readmitted within 90 days decreased from 21.0% (95% CI, 0.12–0.33), to 6.9% (95% CI, 0.01–0.23).

**Conclusion:**

The full standardization was effectively implemented for rectal cancer patients undergoing robot-assisted RRR. The risk of AL, LOS and readmission decreased during the study period. A team focus on high-reliability and peri-operative complications can improve patient outcomes.

**Supplementary Information:**

The online version contains supplementary material available at 10.1186/s12893-022-01809-3.

## Background

Colorectal cancer is the third most common cancer worldwide, and rectal cancer accounts for approximately one third of the total colorectal cancer incidence [1]. In 1982, RJ Heald introduced the concept of total mesorectal excision (TME) [2]. TME is fundamental in modern sphincter-preserving rectal resection with an anastomosis i.e. restorative rectal resection (RRR). RRR was originally performed by an open approach, but with the introduction of laparoscopic surgery and later robot-assisted surgery, many rectal cancer centers perform RRR with robot-assisted approach. Minimally invasive surgery has proven to be oncologically comparable to open approach [3–7]. Furthermore, it has been demonstrated that patients undergoing minimally invasive RRR have an improved postoperative recovery compared to patients undergoing surgery with open approach [7, 8].

Despite improvements in surgical treatment, one major issue continues to challenge colorectal surgeons worldwide—the risk of anastomotic leakage (AL). AL is negatively associated with overall survival [9, 10], increased risk of recurrence [11], and is negatively associated with long-term functional outcome [12]. Studies have reported the risk of AL to vary from 0 to > 20%, although various definitions of AL challenge comparison between the studies [9, 13–18]. Standardization of surgery has been suggested to improve short-term outcome for patients as well as oncological and functional outcome [19–23]. However, standardization has not been implemented in many rectal centers including our department. In recognition of a persistently high risk of AL following robot-assisted RRR [24], our department decided to implement a standardized surgical approach with the aim of decreasing the risk of AL. Such standardization of the surgical technique in RRR, with one or more specific surgical steps, has been reported using laparoscopic approach [19–22, 25], but not with robot-assisted approach. Furthermore, the studies have not described whether it was possible to implement the full standardization or whether various surgeons with different levels of experience, surgical unpredictability and heterogeneity of patients hindered implementation of the full standardization.

Our department decided to implement a specific standardized technique for RRR which has previously been applied at Cleveland Clinic, Ohio [19] and has resulted in low AL rates. The standardized technique was introduced by Professor Dr. Conor P. Delaney (CPD) and consists of a number of specific surgical steps.

The primary aim of this study was to evaluate the process of implementing a standardized technique, for all rectal cancer patients undergoing robot-assisted RRR to reduce variability in outcomes. Specifically, besides evaluating the implementation process, we aimed to evaluate the risk of anastomotic leakage during and after the implementation as well as length of hospital stay and the readmission rate within 90 days.

## Methods

### Design and settings

This single centre cohort study was conducted on all rectal cancer patients undergoing intended minimally invasive RRR at Aarhus University Hospital (AUH) between the 16th of October 2017 and the 14th of October 2020. AUH is one of two primary referral centres managing all rectal cancer surgery in Central Denmark Region, with a population of approximately 1.3 mill inhabitants. The annual volume of RRR is in the range of 60–75 at AUH [26]. Peri-operatively, all patients underwent a multimodal fast-track regimen [27]. The study was approved by The Danish Data Protection Agency (j.nr. 1-16-02-11-18), Danish Patient Safety Authority (case number 3-3013-3006/1), and Central Denmark Region (case number 1-45-70-47-20).

### Cohort

All patients with rectal cancer located within 15 cm from the anal verge undergoing intended minimally invasive RRR were included. Patients who underwent primary open approach were excluded.

### Surgical setting

All resections were elective. The surgical strategy for each patient was decided at a multi-disciplinary team meeting including specialists in colorectal cancer surgery, radiology, oncology, and pathology. Prior to surgery patients received moviprep^®^ as mechanical bowel preparation, but patients did not receive oral antibiotics. All patients were administered a single dose of intravenous antibiotics at the beginning of surgery. The surgical approach was robot-assisted laparoscopy as first choice. In case of lack of capacity for robot-assisted surgery, laparoscopic surgery was performed. Open surgery was conducted only in case of advanced tumour requiring resection beyond the mesorectal fascia and were excluded in this study. Depending on tumour location measured as distance from the anal verge (tumour height), PME or TME was planned [28]^29^. As routine, a defunctioning loop-ileostomy was created only when TME was performed. When the distal rectum was transected with an endoscopic stapling technique, the surgeon used a Da Vinci stapler, 60 mm or 45 mm.

All surgeons were proficient in both robot-assisted and laparoscopic surgery. At least one certified colorectal consultant surgeon participated in each operation. When a training fellow performed the operation, a certified colorectal surgeon supervised the operation.

### The intervention

The standardization of surgical steps for robot-assisted RRR at AUH was implemented from the 16th of October 2017 and included the following steps:

1. Central ligation of the inferior mesenteric artery (IMA) proximally to the left colic artery
2. Central ligation of inferior mesenteric vein (IMV) just below the pancreas
3. Mobilization of the splenic flexure
4. Transection of the anococcygeal raphe (only for TME)
5. Perpendicular transection of rectum
6. Confirmation of arterial bleeding from an arcade artery of the distal colon before performing the anastomosis
7. Perforation of the spike from the circular stapler straight through the stapler line or just in front of the stapler line (optional)

### The process of implementing the standardization

In the Spring of 2017, Professor Dr. CPD from Cleveland Clinic, Ohio, USA, visited AUH. In a one-hour morning session, colorectal surgeons from AUH were taught and instructed in the standardization by CPD. The same day, CPD participated in two RRRs to evaluate a few of the surgeons. Four colorectal surgeons were supervised by CPD. CPD constructed a detailed description (Additional file 1) on how to perform a standardized RRR with anastomosis. The consultant surgeons at AUH discussed the description and agreed on the final standardization as described above. However, the surgeons realized that it could be difficult to determine if step 7 was performed. Therefore, step 7 was made optional.

The standardization was mandatory for all intended minimally invasive RRR for rectal cancer patients. However, surgeons were allowed to deviate from it. Immediately after surgery, the surgeon registered on a written form if steps 1–6 were performed (Additional file 2). In case of deviation from the standardization, the surgeon had to list the reason for the deviation on the written form. All anastomoses were circular double stapled.

In a two-week period in January 2018, two AUH surgeons were on on-site visit at Cleveland Clinic to observe the performance of the standardized RRR.

Throughout the study period, the surgeons at AUH evaluated the standardization and the risk of AL at two meetings in August 2018 and February 2020, respectively. Based on persistently high AL rate, the meetings led to the following changes:

1. From September 2018: Preoperative systemic dexamethasone administration was prohibited. Transection of the rectum with an endoscopic stapler was prohibited in males having TME, and transection should be performed through a Pfannenstiel incision and using a Contour or TA stapler.
2. From March 2020: Transection of the rectum with an endoscopic stapler was allowed in PME only, and if the surgeon anticipated using only one or two firings/magazines. Otherwise, transection was performed through a Pfannenstiel incision and a Contour or TA stapler.

Based on the two meetings, the study was divided into three periods: Period 1 (October 2017–August 2018), period 2 (September 2018–February 2020), and period 3 (March 2020–October 2020).

### Data sources

The written form (Additional file 2), which was filled in by the surgeons immediately post-surgery, included the following data: Name of the surgeons, times of start and end of surgery, intraoperative bleeding, patient weight and height, use of Indocyanine green (ICG), type of stapler used for transection of the rectum, number of firings (if the stapler was endoscopic), name of circular stapler, type of anastomosis (i.e. side-to-end, end-to-end, other), besides information on whether steps 1–6 of the standardization were performed (yes/no), and if not, the reasons for the deviation. In patients with no registered form or missing data, the steps in the standardization were retrieved from medical records by JDE (who did not participate in or performed any of the RRRs). JDE entered the form data in the Redcap database [30]. Data on ASA, smoking, alcohol intake, neoadjuvant therapy, tumour height, preoperative systemic dexamethasone administration, defunctioning stoma, length of hospital stay (LOS), AL within 30 days, readmission within 90 days, and death within 90 days were retrieved from medical records (reviewed by JDE).

### Outcome

The evaluation of implementation was based on the complete usage of the standardization. If all of steps 1–6 were followed (step 4 for TME only), implementation of the standardization was defined as ‘yes’. If deviation from at least one of the six steps occurred, implementation of the standardization was defined as ‘no’.

AL was defined as a defect of the intestine in relation to the anastomosis leading to communication between the intra and extra luminal compartments [31]. A pelvic abscess without a proven defect in the stapler line was included as an AL. Severity of AL was graded according to the definition from The International Study Group of Rectal Cancer [31]. Only symptomatic AL was included; hence, grade A AL was not included. Patients were diagnosed with AL by computed tomography with rectal contrast, endoscopy, or reoperation because of clinical symptoms and/or biochemical analyses, suggestive of AL according to clinical practice. Patients were registered as readmitted, if they were readmitted one or more times within 90 days following discharge after surgery. The total number of days readmitted was defined as all days registered as an in-hospital patient within 90 days following discharge after surgery.

### Statistics

Statistical analysis was performed in Stata/SE 15.1 (StataCorp LLC, College Station, Texas, USA). Continuous data were transformed into categorical variables and presented as numbers and percentages. Usage of the standardization was defined as the proportion of RRR procedures in which a full standardization was used, i.e., steps 1–6, among the entire number of minimally invasive RRR procedures. A sub-analysis was performed calculating the proportion of RRR procedures in which a full standardization was used among patients with a registered form. The absolute risk of AL was calculated as proportion of patients undergoing RRR who developed AL within 30 days. The rate of patients readmitted was calculated as proportion of patients readmitted within 90 days. To compare the usage of the surgical standardization, the risk of AL, the LOS, and the rate of patients readmitted between the tree periods, Kruskal–Wallis test was used. *p*-value < 0.05 was considered statistically significant.

## Results

**Fig. 1:**
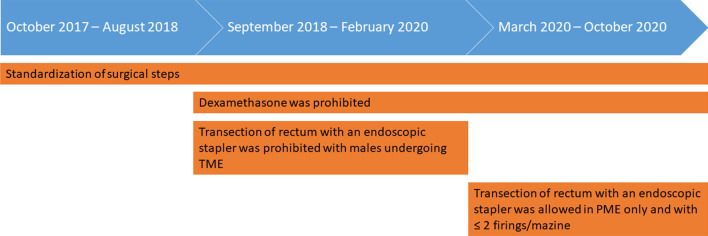
Timeline of the study period, October 16th 2017—October 14th 2020 with the standardized changes throughout the period with rectal cancer patients undergoing intended robot-assisted restorative rectal resection at Aarhus University Hospital. *TME* total mesorectal excision, *PME* partial mesorectal excision

A total of 165 patients with rectal cancer underwent minimal invasive RRR at AUH between the 16th of October 2017 and the 14th of October 2020. Ten different surgeons performed the operations as the primary surgeon. The surgeons were all either certified colorectal surgeons or training fellows in colorectal surgery. Patient characteristics are presented in Table 1. A timeline illustrating the interventions throughout the study period with implementation of the standardization, and the additional changes with prohibition of systemic dexamethasone and limited use of the endoscopic stapler is presented in Fig. 1.

**Table 1:** Characteristics of patients with rectal cancer undergoing robot-assisted restorative rectal resection at Aarhus University Hospital, 2017–2020

	Period 1: October 2017–August 2018	Period 2: September 2018–February 2020	Period 3: March 2020–October 2020
N patients	62	74	29
Sex			
Males	47 (75.8)	43 (58.1)	22 (75.9)
Females	15 (24.2)	31 (41.9)	7 (24.1)
Median, age, years (range)	66 (43–84)	67 (39–91)	61 (45–80)
Median BMI, kg/m^2^ (range)	26 (19–42)	26 (16–43)	26 (20–35)
American Society of Anesthesiology score (ASA)			
1	14 (22.6)	22 (29.7)	11 (37.9)
2	39 (62.9)	45 (60.8)	16 (55.2)
3	9 (14.5)	7 (9.5)	1 (3.4)
Missing	0	0	1 (3.4)
Smoking			
Never	23 (37.1)	41 (55.4)	13 (44.8)
Previous (≥ 8 weeks)	25 (40.3)	17 (23.0)	10 (34.5)
Current	14 (22.6)	16 (21.6)	6 (20.7)
Alcohol intake/week (units)			
0	1 (1.6)	6 (8.1)	6 (20.7)
1–14	56 (90.3)	65 (87.8)	21 (72.4)
15–20	3 (4.8)	2 (2.7)	1 (3.4)
≥ 21	2 (3.2)	1 (1.4)	1 (3.4)
Neoadjuvant (chemo)radio-therapy			
No	55 (88.7)	60 (81.1)	23 (79.3)
Yes	7 (11.3)	14 (18.9)	6 (20.7)
Tumor height (cm from anal verge)			
11–15 cm	34 (54.8)	41 (55.4)	13 (44.8)
6–10 cm	25 (40.3)	26 (35.1)	15 (51.7)
≤ 5 cm from anal verge	3 (4.8)	0	0
Missing	0	7 (9.5)	1 (3.4)
Surgery by certified colorectal surgeon			
No	6 (9.7)	3 (4.1)	1 (3.4)
Yes	56 (90.3)	71 (95.9)	28 (96.6)
Dexamethasone			
No	14 (22.6)	70 (94.6)	28 (96.6)
Yes	48 (77.4)	4 (5.4)	1 (3.4)
Surgical Approach			
Robotic	59 (95.2)	71 (95.9)	29 (100)
Laparoscopic	3 (4.8)	3 (4.1)	0
Converted from minimally invasive			
No	60 (96.8)	73 (98.6)	28 (96.6)
Yes	2 (3.2)	1 (1.4)	1 (3.4)
Resection type			
TME	42 (67.7)	48 (64.9)	16 (55.2)
PME	20 (32.3)	26 (35.1)	13 (44.8)
Missing			
Stapler (type) used for transection of distal rectum			
Robot	52 (83.9)	36 (48.6)	3 (10.3)
EndoGIA	4 (6.5)	3 (4.1)	0
Endoscopic, not specified	1 (1.6)	0	0
Contour	5 (8.1)	34 (45.9)	26 (89.7)
TA	0	1 (1.4)	0
Firings (Endoscopic stapling of distal rectum)			
1	0	3 (7.7)	0
2	47 (82.5)	30 (76.9)	3 (100)
3	7 (12.3)	5 (12.8)	0
4	1 (1.8)	0	0
Missing	2 (3.5)	1 (2.6)	0
Defunctioning stoma for resection type PME			
No	20 (100)	26 (100)	12 (92.3)
Yes	0	0	1 (7.7)
Defunctioning stoma for resection type TME			
No	0	1 (2.1)	0
Yes	42 (100)	47 (97.9)	16 (100)
Mean operation time, min (range)	228 (100–419)	262 (160–504)	281 (185–389)
Resections with a written form registered by the surgeon	58 (93.5)	63 (85.1)	25 (86.2)
Median length of hospital stay, days (range)	6 (2–50)	5 (2–37)	5 (2–26)
Number of patients readmitted	13 (21.0)	7 (9.6)	2 (6.9)
Median length of total number of days as readmitted (range)	5 (1–22)	8 (2–18)	6 (3–9)

Number (percentages), unless indicated otherwise

BMI, body mass index; TME, total mesorectal excision; PME, partial mesorectal excision, TA, thoracoabdominal

**Fig. 2:**
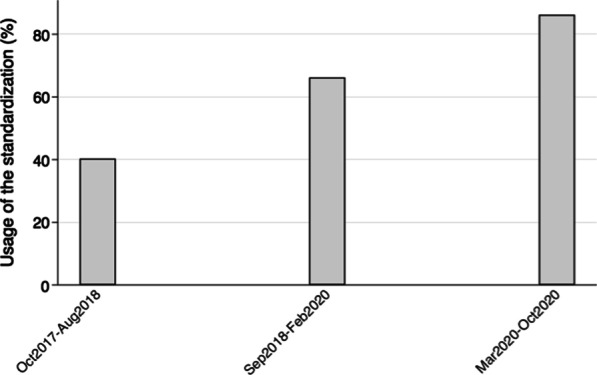
Rectal cancer patients undergoing restorative rectal resection (RRR) with an intended robot-assisted approach at Aarhus University Hospital (AUH), 2017–2020 and with usage of the standardization

A total of 88.5% of the patients had a written form registered by the surgeon immediately after the operation. Table 2 demonstrates the fulfilment of the specific steps in the standardization based on the form registration or medical records. From period 1 to period 3 usage of the standardization increased from 40.3% (95% CI, 0.28–0.54), over 66.2% (0.54–0.77) in period 2 to 86.2% (95% CI, 0.68–0.95) (*p* < 0.001) (Fig. 2). A sub-analysis was performed excluding patients with no registered form, and in this sub analysis usage of the standardization increased throughout the periods from 43.1% (95% CI, 0.30–0.57) to 96.0% (95% CI, 0.80–1.00).

**Table 2:** Patients with rectal cancer undergoing robot-assisted restorative rectal resection with at Aarhus University Hospital, 2017–2020 and the specific steps in the standardization based on the written form registered by the surgeon immediately after the operation or review of medical records. Number (percentages)

	Period 1: October 2017–August 2018	Period 2: September 2018–February 2020	Period 3: March 2020–October 2020
Central transection of IMA (high ligation)			
No	23 (37.1)	10 (13.5)	1 (3.4)
Yes	34 (54.8)	55 (74.3)	25 (86.2)
Missing	5 (8.1)	9 (12.2)	3 (10.3)
Central transection of IMV			
No	23 (37.1)	4 (5.4)	0
Yes	35 (56.5)	66 (89.2)	28 (96.6)
Missing	4 (6.5)	4 (5.4)	1 (3.4)
Mobilization of splenic flexure			
No	20 (32.3)	5 (6.8)	0
Yes	41 (66.1)	69 (93.2)	29 (100)
Missing	1 (1.6)	0	0
Transection of the anococcygale ligament (TME)			
No	0	0	0
Yes	40 (95.2)	41 (85.4)	15 (93.8)
Missing	2 (4.8)	7 (14.6)	1 (6.3)
Perpendicular transection of rectum			
No	0	0	0
Yes	56 (90.3)	61 (82.4)	26 (89.7)
Missing	6 (9.7)	13 (17.6)	3 (10.3)
Arterial bleeding of arcade artery			
No	2 (3.2)	0	0
Yes	59 (95.2)	72 (97.3)	28 (96.6)
Missing	1 (1.6)	2 (2.7)	1 (3.4)

*IMA* inferior mesenteric artery, *IMV* inferior mesenteric vein, *TME* total mesorectal excision

AL occurred in 24 patients (14.5%) (Table 3). For patients undergoing PME and TME the AL rate was 3.4% and 20.8%, respectively. AL was verified with computed tomography with rectal contrast extravasation and/or endoscopy with a defect in 22 patients. Two patients with AL had a pelvic abscess without a proven defect in the stapler line. From period 1 to period 3 the risk of AL decreased from 21.0% (95% CI, 0.12–0.33), over 12.2% (95% CI, 0.06–0.22) in period 2 to 6.9% (95% CI, 0.01–0.23), respectively (*p* = 0.15). LOS decreased from 6 days (range 2–50) over 5 days (range 2–37) (*p* = 0.55), to 5 days (range 2–26) (*p* = 0.55). The rate of patients readmitted within 90 days decreased from 21.0% (95% CI, 0.12–0.33), over 9.6% (95% CI, 0.39–0.19), to 6.9% (95% CI, 0.01–0.23) (*p* = 0.08).

**Table 3:** The proportion of anastomotic leakage with rectal cancer patients undergoing robot-assisted restorative rectal resection at Aarhus University Hospital, 2017–2020

	Period 1: October 2017–August 2018	Period 2: September 2018–February 2020	Period 3: March 2020–October 2020
Anastomotic leak	13/62 21.0% (0.12–0.33)	9/74 12.2% (0.06–0.22)	2/29 6.9% (0.01–0.23)
Type B (n)	11	8	2
Type C (n)	2	1	0

Number/total and proportion (95% CI), unless indicated otherwise

## Discussion

In this prospective cohort study of patients undergoing robot-assisted RRR, we evaluated the process of implementing a standardization of surgical steps aiming to reduce the risk of AL. We found that the usage of the standardization increased statistically significant throughout the study period. Additional changes were made with prohibition of systemic dexamethasone administration and limited use of endoscopic stapler to transect the rectum. The changes were launched immediately after each decision, and they were followed almost completely. During the study period, the risk of AL, LOS and the rate of patients readmitted decreased, however not significantly.

This study clearly indicates that it is feasible to implement a standardization of a surgical procedure at a department employing several surgeons. There is no clear evidence of the effect of the individual steps in the standardization and the additional changes on the risk of AL. However, there is evidence that the risk of AL is multifactorial [32, 33]. Therefore, it seems important to standardize treatment as much as possible. This will potentially decrease the risk of confounding in future studies investigating the risk of AL after RRR. If various centers agree on the same standardized treatment, this could also decrease the heterogeneity when performing multicenter studies. The current study is the first of its kind to describe an implementation of technical aspects of robot-assisted RRR, and whether it was feasible in every operation and with several surgeons. However, implementation of other regimens has been published. Conn et al. described important factors for successful implementation of Enhanced Recovery After Surgery (ERAS)-program in colorectal surgery [34]. They experienced that belief in the importance of the program and making the program highly visible were key elements for a successful implementation. We experienced similar important factors for the implementation of the standardization. In particular, three factors were found to be important; 1: Our department had experienced a period of time with a high occurrence of AL which made the surgeons highly motivated for adjustments that could potentially lower the risk of AL. 2: All surgeons at the department participated in the planning of the standardization, and eventually agreed upon the standardization. 3: Data regarding usage of the standardization and the risk of AL were presented at the two evaluation meetings which made it visible, whether the standardization was followed or not. Other approaches could have potentially accelerated the implementation with closer evaluation of each individual surgeon’s usage of the standardization. However, we wanted to create a realistic clinical setting, which could be transferred to other colorectal centers.

We implemented the standardization to lower the risk of AL and supplemented with additional changes (prohibition of systemic dexamethasone administration, limiting the use of endoscopic stapler devices), and the subsequent decline in the risk of AL was successful. However, the study is underpowered to perform multivariate analysis or to draw any valid conclusions on those topics. LOS and the rate of readmitted patients decreased as the risk of AL decreased. The criteria for discharging patients remained unchanged in the study period. A decrease in LOS and the rate of readmitted patient will benefit the patients, and furthermore, it will have a positive impact on health economics.

### The elements in the standardized approach and the association with AL

The different steps in the standardized approach described in this study and their association with AL have been subjects to debate since the procedure was first described.

The level of ligation of IMA (high tie versus low tie) and the association with AL have been described in randomized controlled trials, meta-analysis and population-based cohort studies, and there is no consensus whether high tie or low tie should be preferred to decrease the risk of AL [35–37]. Regarding splenic flexure mobilization (SFM) it is debated whether to perform routine SFM or selective SFM when tension on the anastomosis occurs. Advocates for routine SFM argue that it is not possible to assess the tension on the anastomosis because no formal measurement of anastomotic tension exist [38]. Furthermore, they argue that it is preferable to perform a standardized procedure, and it is most optimal to perform SFM before doing the pelvic operation [38]. Advocates for selective SFM argue that SFM increases operation time, and increases the risk of iatrogenic damage to the spleen [39], and there is no need to undertake an additional surgical step, if the anastomosis can be safely constructed without undue tension [40]. Transection of the anococcygeal raphe (only for TME), perpendicular transection of rectum, and confirmation of arterial bleeding from an arcade artery of the distal colon before performing the anastomosis were all steps included in our standardization based on a general consideration on how to perform a safe anastomosis.

Along with implementation of minimally invasive approach, it has become possible to transect the distal rectum with an endoscopic stapler, which is an alternative stapler type to the ones used during the classical open approach. Due to stapling in the deep narrow pelvis and a suboptimal cutting angle, the need for multiple magazines/firings often occurs. Several studies have demonstrated that the risk of AL increases with use of the endoscopic stapler and the number of magazines/firings. In particular, when the number of magazines/firings exceeds 2 [17, 33, 41, 42]. This study clearly demonstrated that it was feasible for the surgeons to follow the instructions to perform stapling of the distal rectum with an endoscopic stapling technique with maximum two magazines/firings or otherwise to choose an open stapling technique.

There were a few study limitations. The study cohort was small; the surgeons could have been prone to the Hawthorne effect [43], which could have made the surgeons perform surgery with other routines than those planned. However, this study aimed to evaluate the implementation of the standardization, hence the Hawthorne effect would not influence the results. Furthermore, the Hawthorne effect and attention to detail could possibly improve the team focus and patient outcomes. It was not possible to make any conclusions regarding AL rate due to the small study cohort. Finally, there was no control with the written form filled out by the surgeon, and incorrect registration could have occurred—intentionally or unintentionally. Incorrect registration of the written form would not have influenced the risk of AL.

## Conclusion

In conclusion, this study describes the evaluation of implementing the standardization for all rectal cancer patients undergoing robot-assisted RRR at AUH. Additional changes to the perioperative protocol were made and effected immediately, and the risk of AL decreased during the study period. We recommend testing the standardization in a larger setting. A team focus on high-reliability and peri-operative complications can improve patient outcomes.

## Supplementary Information

**Additional file 1.** A detailed description from Dr. Conor P. Delaney on how to perform a standardized restorative rectal resection.

**Additional file 2.** The written form that the surgeons registered immediately after surgery. TME, total mesorectal excision; PME, partial mesorectal excision.

## Data Availability

The data that support the findings of this study are available from Central Denmark Region but restrictions apply to the availability of these data, which were used under license for the current study, and so are not publicly available. Data are however available from the authors upon reasonable request and with permission of The Danish Data Protection Agency, Danish Patient Safety Authority, and Central Denmark Region.
