# Supplementary material for: Implementation of a standardized surgical technique in robot-assisted restorative rectal cancer resection: a single center cohort study
Source: BMC Surg. 2022 Oct 13;22:360. doi: 10.1186/s12893-022-01809-3 (PMC9563459; doi:10.1186/s12893-022-01809-3)
Supplement: Supplementary file 1 — Additional file 1. A detailed description from Dr. Conor P. Delaney on how to perform a standardized restorative rectal resection. [file 12893_2022_1809_MOESM1_ESM.docx]

Supplementary material 1. Detailed description from Professor Dr. Conor P. Delaney on how to perform a restorative rectal resection.

Resection phase:

1. During the IMA dissection, stay anterior to Toldt’s fascia. This helps protect the periaortic nerves and potentially sexual function.

2. Stay between the visceral (mesorectal) and parietal (pelvic sidewall / Denonvillier’s) layers of fascia. This helps protect the nerves, and other sidewall structures.

3. Preserve the presacral fascia unless there is direct posterior invasion – may help reduce potential for significant bleeding in other cases.

4. Try and follow the mesorectal fascia laterally. I think you can apply more medial traction to visualize. Changing sides more frequently may help, but I do not think the platform should dictate the operation.

5. Mobilize down to the anorectal junction. At this level there is almost no residual mesorectum. It is an easier transection, and I am not aware of any functional benefit of saving 2-4cm of rectum. Dividing a few cm higher ends up being a more difficult transection level.

Transection phase:

1. Always do a rectal exam. Always get a 5cm margin if you can, and accept 2cm for stapled. If margin less than this I would do an inter-sphincteric resection, accepting 1cm after a pre-operative discussion for well-differentiated tumors, younger motivated patients, etc.

2. Always feel the stapler is perpendicular to the rectum by digital exam before firing, and this allows measurement of the distal margin.

3. Never leave a jagged, distal staple line. Always make the distal staple line look like a single stapler has been used, so there are no ischemic triangles distally.

Mobility and Flexure:

1. I would recommend a high ligation of the IMA in all cases for oncologic and reach reasons. The left colic is then divided off the IMA, preserving the bifurcation of the left colic. The inferior mesenteric vein is taken at this level.

2. Personally, I mobilize the flexure for all low rectal anastomoses. By review of the literature this is optional, but I would recommend a very low threshold for taking the flexure. This decision needs to be taken before specimen extraction. If the flexure is taken, the IMV is taken near the tail of the pancreas. The transverse colon mesentery is mobilized to near the midline, or midline is some cases.

3. Mobility is assessed for the descending colon and again assessed after rectal transection and before specimen removal.

4. If the robot does not facilitate this, I would think that is a reason to use laparoscopy and not robotics. The platform should never dictate the operation.

Anastomosis Phase:

1. Make sure there is a strong pulse bleeding in the marginal vessel before choosing the site for a purse-string.

2. Consider a second purse string if the first one is at all loose.

3. Return the bowel to the abdomen and make sure it lies easily in the pelvis.

4. Bring the spike out through or immediately beside the staple line, so that the EEA staple line resects a piece of the transverse line.

5. Make sure the neo-rectum lies very easily in the pelvis, with so little tension that it follows the curve of the sacrum.

6. Make sure the donuts are intact full-thickness – not just mucosa.
