# Supplementary material for: Implementation of a standardized surgical technique in robot-assisted restorative rectal cancer resection: a single center cohort study
Source: BMC Surg. 2022 Oct 13;22:360. doi: 10.1186/s12893-022-01809-3 (PMC9563459; doi:10.1186/s12893-022-01809-3)
Supplement: Supplementary file 2 — Additional file 2. The written form that the surgeons registered immediately after surgery. TME, total mesorectal excision; PME, partial mesorectal excision. [file 12893_2022_1809_MOESM2_ESM.docx]

MINIMAL INVASIV OPERATION

Dato____________

label

**Operatør** (navn) __________________________+ _____________________+___________________

**Adgang** (initiale) Robot-assisteret:_____ Laparoskopisk:_______

**Konverteret**: Ja____ Nej_____

**OP-type** (sks-kode + navn på operation)

1.______________________________

2.______________________________

3.______________________________

4.______________________________

5.______________________________

**OP-start** (kl.): _______:______ **OP-slut** (kl): _______:______

**Intraoperativ blødning** (ml):__________________

**Vægt** (kg):___________ **Højde** (cm):____________

**ICG (Firefly) anvendt**: Ja ______ Nej________ Givet kl.:___________

Anvendt til vurdering af: Rectum:_____ Colon:________ Tyndtarm:_________

Andet (angiv):______________

Anvendt i relation til anastomose: Før____ Efter____

Medførte ICG ændringer: Ja ______ Nej________

Angiv ændringer: ____________________________________________


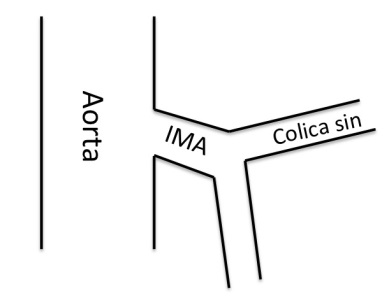
Kun for rektumresektion med anastomose:

Deling af IMA:

Centralt (high tie):________ Efter afgang af a.col.sin (low tie):_______

Central deling af IMV (under pancreas): Ja ______ Nej________

Venstre flexur mobiliseret: Ja ______ Nej________

Hvis nej, angiv årsag:____________________________________

Deling af anococcygale ligament: Ja ______ Nej________

Hvis nej, angiv årsag:____________________________________

Perinealt tryk: Ja ______ Nej________

Rektum delt vinkelret: Ja ______ Nej________

Hvis nej, angiv årsag:____________________________________

Anvendt tværstapler: Robot-stapler____ Contour____ TA-45____ EndoDrive: 60 mm___ 45 mm___ 30 mm___ Anden______ (angiv)_______________

Antal firings______

Magasin Grønt:_____ Purple:_____ Anden______ (angiv)__________

Sprøjtende blødning fra arkadearterien: Ja ______ Nej________

Hvis nej, er der så re-reseceret oralt: Ja ______ Nej________

Hvis nej, angiv årsag:____________________________________

Anvendt cirkulær stapler: Medtronic/Covidien_____ Anden______ (angiv)_______________

Placering af spikens perforation (flere krydser er mulig): Foran staplerlinjen_____ Bag staplerlinjen________

Gennem staplerlinjen___ I hjørne______

Orientering af anastomose: Side-to-end____ End-to-end______ Håndsyet kolo-anal_____ Anden______ (angiv)_______________
